# Technology-Assisted Patient Access to Clinical Information: An Evaluation Framework for Blue Button

**DOI:** 10.2196/resprot.3290

**Published:** 2014-03-27

**Authors:** Timothy P Hogan, Kim M Nazi, Tana M Luger, Daniel J Amante, Bridget M Smith, Anna Barker, Stephanie L Shimada, Julie E Volkman, Lynn Garvin, Steven R Simon, Thomas K Houston

**Affiliations:** ^1^Center for Healthcare Organization and Implementation Research (CHOIR)eHealth Quality Enhancement Research Initiative (QUERI), National eHealth QUERI Coordinating CenterBedford, MAUnited States; ^2^Division of Health Informatics and Implementation ScienceDepartment of Quantitative Health SciencesUniversity of Massachusetts Medical SchoolWorcester, MAUnited States; ^3^Veterans and Consumers Health Informatics OfficeOffice of Informatics and Analytics, Veterans Health AdministrationUS Department of Veterans AffairsWashington, DCUnited States; ^4^Center of Innovation for Complex Chronic Healthcare (CINCCH)Spinal Cord Injury Quality Enhancement Research Initiative (QUERI)Hines VAMCChicago, ILUnited States; ^5^Program in Health Services ResearchStritch School of MedicineLoyola University ChicagoMaywood, ILUnited States; ^6^Department of Health Policy and ManagementSchool of Public HealthBoston University School of Public HealthBoston, MAUnited States; ^7^Section of General Internal MedicineVA Boston Healthcare SystemBoston, MAUnited States; ^8^The Heller School for Social Policy and ManagementBrandeis UniversityWaltham, MAUnited States; ^9^Division of General Internal Medicine and Primary CareBrigham and Women’s HospitalBoston, MAUnited States

**Keywords:** personal health record, United States Department of Veterans Affairs, patient access to records

## Abstract

**Background:**

Patient access to clinical information represents a means to improve the transparency and delivery of health care as well as interactions between patients and health care providers. We examine the movement toward augmenting patient access to clinical information using technology. Our analysis focuses on “Blue Button,” a tool that many health care organizations are implementing as part of their Web-based patient portals.

**Objective:**

We present a framework for evaluating the effects that technology-assisted access to clinical information may have on stakeholder experiences, processes of care, and health outcomes.

**Methods:**

A case study of the United States Department of Veterans Affairs' (VA) efforts to make increasing amounts of clinical information available to patients through Blue Button. Drawing on established collaborative relationships with researchers, clinicians, and operational partners who are engaged in the VA’s ongoing implementation and evaluation efforts related to Blue Button, we assessed existing evidence and organizational practices through key informant interviews, review of documents and other available materials, and an environmental scan of published literature and the websites of other health care organizations.

**Results:**

Technology-assisted access to clinical information represents a significant advance for VA patients and marks a significant change for the VA as an organization. Evaluations of Blue Button should (1) consider both processes of care and outcomes, (2) clearly define constructs of focus, (3) examine influencing factors related to the patient population and clinical context, and (4) identify potential unintended consequences.

**Conclusions:**

The proposed framework can serve as a roadmap to guide subsequent research and evaluation of technology-assisted patient access to clinical information. To that end, we offer a series of related recommendations.

## Introduction

### The Blue Button

Patient engagement is associated with desirable outcomes, including increased satisfaction with care, improved well-being, and better medical adherence [1-4]. Critical to engagement is the ability for patients to access and manage their personal health information [4,5]. Information has long been understood as an essential resource for managing health problems [6], and interacting with health-related information is an integral component of that work [7,8]. “Personal health information management” refers to the activities that support individuals’ access, organization, and use of information pertaining to their own health [9,10]. “Clinical information”, a core subset of personal health information most often stored in health records, is patient-related information that can be used to support decisions and facilitate tasks related to a patient’s care. Typically, this includes, but is not limited to, doctor’s notes, patient health history and status, medication lists, lab results, and data regarding usage of services.

Historical policies, workflows, and technologies have often limited patient access to clinical information. Traditionally, file cabinets, and later, electronic health records (EHRs), were the domains of clinical information, secure vaults that were inaccessible to patients except upon written request, and typically after a delay and the payment of requisite fees. Today, however, many public and private health care organizations are exploring ways to facilitate patient access to and exchange of personal health information, including clinical information. Policies are changing, and legislation has been signed into law to support increased patient access to clinical information [11-13]. EHR-connected (ie, tethered) patient portals and personal health records (PHRs) [14,15] are now being positioned as a means to achieve the patient-centric objectives of Meaningful Use, including direct patient access to clinical information [16,17].

In this paper, we examine the movement toward augmenting patient access to clinical information-using technology. Our analysis focuses on “Blue Button,” a tool that many health care organizations are implementing as part of their Web-based patient portals. The Blue Button concept originated at a January 2010 meeting of the Markle Foundation Consumer Empowerment Workgroup [18], in which representatives from government and private industry envisioned that adding a “big blue button” to patient portals would enable patients to have more direct access to view and download their clinical information. The United States Department of Veterans Affairs (VA) partnered with the Department of Defense and Centers for Medicare & Medicaid Services, mobilizing to release the first Blue Button on each agency’s beneficiary portal within the next 8 months. Since that time, Blue Button has evolved from a basic idea to a national movement to put health information into the hands of consumers in a way that they can use it. The Office of the National Coordinator for Health Information Technology, part of the Department of Health and Human Services, has embraced the concept and set multiple supporting initiatives in motion, including the Blue Button Pledge to inspire industry commitment, various challenges and contests to optimize information presentation through Blue Button, and efforts to improve consumer awareness and to articulate a vision for Blue Button expansion. Over 450 organizations have now taken the Blue Button Pledge and committed themselves to advancing patient access to and use of personal health information as a way to improve health and the delivery of care [19,20]. Support for Blue Button was further underscored in a Markle Foundation survey that found that 70% of patients and 65% of doctors agree that patients should be able to download and keep copies of their own clinical information [21]. Providing patients the ability to view, download, and transmit their health information is also an objective of Stage II Meaningful Use [22].

Using the VA as a case study, we characterize the experiences that one organization has had as it mobilized to make increasing amounts of clinical information available to its patients. Building from this foundation, we present a framework to evaluate the effects that access to clinical information may have on stakeholder experiences, processes of care, and outcomes. We conclude with a series of recommendations to guide future research in this rapidly evolving area.

### Making Clinical Information Accessible

Facilitating patient access to clinical information has been discussed as a means to improve the transparency and delivery of care as well as interactions between patients and health care providers [5,23]. Although earlier research showed that patients were not only eager to access clinical information, but also quite capable of understanding the information that they obtained [24], persistent concerns have remained among physicians that such access could result in patient harm [25,26]. These concerns center on both the medical jargon often present in clinical information and the inclusion of diagnoses and other content that might be viewed unfavorably by patients [24].

The movement toward making clinical information more accessible to patients has been framed as part of broader efforts to promote effective health information exchange across organizations [27]. It is also frequently discussed in the context of eHealth, a subdomain of consumer health informatics that involves the use of information technology to deliver health information and services to patients and family members [28-30]. Improved access to and sharing of clinical information is anticipated to enhance patient-provider communication, provider-provider communication in disparate settings, patient self-management practices, and to facilitate appropriate usage of services [31,32]. Patients value having increased access to their information and see it as a way to better understand and become more involved in their health [33-36].

### Open Notes

Most recently, the “Open Notes” Project provided a new assessment of outcomes associated with patient access to clinical information. Studying primary care practices at three medical facilities (Beth Israel Deaconess Medical Center in Massachusetts, Geisinger Health System in Pennsylvania, and Harborview Medical Center in Washington), DelBanco and colleagues [37] found that providing Web-based access to the notes that physicians wrote following a patient visit was perceived positively by the majority of the patients in the study: 77% to 87% across the three sites reported that access to their doctor’s notes helped them to feel more in control of their care. Despite physician’s initial concerns about patient access to notes causing unnecessary worry [32], 99% of the participating physicians wanted access to continue at the end of the study. Furthermore, few of the participating physicians reported that the practice of Open Notes negatively impacted their workload, and none elected to stop providing access to their notes at the conclusion of the study [37]. At one of the three study sites (Harborview Medical Center) a higher proportion of patients (14%) described their notes as confusing [32]. As we explore below, this finding raises important questions about patient population characteristics and the clinical context in which increased access to clinical information transpires.

## Methods

### Case Study: Blue Button in VA

Drawing on established collaborative relationships with researchers, clinicians, and operational partners, we examined the efforts underway in the VA to make increasing amounts of clinical information available to patients through Blue Button. We assessed existing evidence and organizational practices through key informant interviews with VA researchers and representatives from relevant VA program offices, reviewed historical documents, usage reports, data documentation, and other materials describing Blue Button, and conducted an environmental scan of the published literature and websites of other health care organizations to contextualize our findings.

Veterans using the VA PHR patient portal, My Health***e***Vet, have consistently provided feedback that they value increased access to their medical records [38]. In response, VA Blue Button was added to My Health***e***Vet in August 2010, enabling Veterans to view, print, or download a single electronic file with all of their available personal health information. Registered portal users can include self-entered information in their Blue Button files, while VA patients who also complete an identity-proofing process can include both self-entered information and clinical information extracted from the VA EHR. Table 1 presents a comprehensive list of the types of information available to identified-proofed VA patients through VA Blue Button.

**Figure 1:**
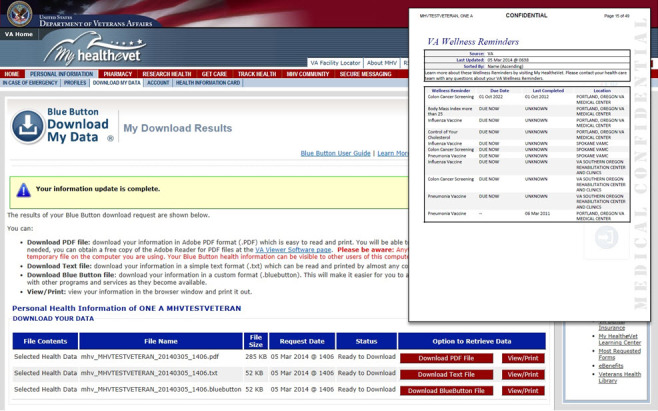
VA Blue Button download results screen and blue button file.

Veterans using VA Blue Button can choose to view and print their information from a Web browser window, or download their information in portable document format (PDF), as a plain text file, or as a Blue Button text file intended to support use with other electronic applications. Veterans can tailor the Blue Button file by selecting specific date ranges and/or specifying the types of information that they wish to include. Figure 1 shows the VA Blue Button download results screen within the My Health***e***Vet PHR portal; the inset shows the format of a Blue Button file.

Expansions of the clinical information available through VA Blue Button have been released incrementally. Some information is accessible after a brief delay to allow time for health care providers to communicate directly with patients, for example to discuss abnormal test results. In January 2013, the VA joined the OpenNotes Initiative, sponsored by the Robert Wood Johnson foundation [37], and now offers patients open access to their clinical progress notes authored from January 1, 2013 forward. The VA also introduced patient access to a Continuity of Care Document through Blue Button; a standards-based health summary available in extensible markup language (XML) and PDF file formats. All of these efforts build on the success of the My Health***e***Vet Pilot Program [35], align with specifications for Meaningful Use, and reflect the VA’s commitment to patient-centered care.

**Table 1:** Personal health information, including clinical information available through VA Blue Button.

Type of information		Description	When available	Date range
**Patient self-reported information**				
	Activity journal	Daily exercise and activity log	Immediately	User selected
	Allergies	History of allergies including severity, reaction, diagnosis, and comments	Immediately	All
	Demographics	Personal information entered during account registration or profile updates, emergency contacts	Immediately	All
	Family health history	Family member’s health history and events that may affect health	Immediately	All
	Food journal	Daily food intake to monitor diet or control weight	Immediately	User selected
	Health care providers	Information pertaining to caregivers and health care providers	Immediately	All
	Health insurance	Information about health insurance coverage and policies	Immediately	All
	Immunizations	Immunization date, method used, and any reactions	Immediately	All
	Labs and tests	Information about lab tests performed and test results	Immediately	User selected
	Medical events	History of illnesses, accidents, or other events	Immediately	All
	Medications and supplements	Medications, over-the-counter drugs, herbals, and supplements	Immediately	All
	Military health history	Military health history, potential exposures, and treatments	Immediately	All
	My goals (current goals and completed goals)	Set individualized, personally relevant recovery goals and track progress toward achieving these goals	Immediately	Current goals: all completed goals: user selected
	Treatment facilities	Medical treatment facilities and locations	Immediately	All
	Vitals and readings	Common health measures (eg, blood pressure, blood sugar, pain, etc.)	Immediately	User selected
**VA EHR** ^a^ **information**				
	VA allergies^b^	Recorded allergies and adverse reactions	Immediately	All
	VA admissions and discharges^b^	Admissions and discharges including comprehensive discharge summaries	Discharge Summary only: 3 days after completed	User selected
	VA appointments^b^	Two years past and all future VA appointment details	Immediately	All
	VA demographics^b^	Demographic information from VA treating facilities in the last 3 years	Immediately	All
	VA electrocardiogram (EKG) reports^b^	A list of EKG studies performed at VA treating facilities	Immediately	User selected
	VA immunizations^b^	History of recorded immunizations along with any reactions	Immediately	All
	VA laboratory results^b^	Results of chemistry, hematology, and microbiology lab tests	3 days after results verified	User selected
	VA medication history	History of VA medication refills	Immediately	User selected
	VA notes^b^	All completed progress notes from January 1, 2013 forward	3 days after Note completed	User selected
	VA pathology reports^b^	Surgical pathology, cytology, and electron microscopy study results	14 days after report completed	User selected
	VA problem list^b^	List of active health issues and conditions	3 days after entry	All
	VA radiology reports^b^	Results of radiology and other imaging studies	3 days after report verified	User selected
	VA vitals and readings^b^	Blood pressure, pulse, body temperature, weight, etc.	Immediately	User selected
	VA wellness reminders^b^	Patient friendly clinical reminders for preventive services	Immediately	All
**Department of defense information**				
	Military service information^b^	Historical record of military service including position and rank codes	Immediately	All

^a^Electronic health record

^b^Requires My Health***e***Vet account authentication

### VA Blue Button Usage to Date

Through January, 2014, there were 2,127,462 VA patient registrants with the My Health***e***Vet PHR portal (a 37.57% penetration rate among all VA patients in fiscal year 2013), and 1,456,807 VA patients who had completed the identity proofing process for the portal (a 25.73% penetration rate among all VA patients in fiscal year 2013). Over 955,800 unique registered users had submitted download requests through VA Blue Button, downloading over 5.7 million files [39]. As we describe below, it is important to recognize that Blue Button is one of many technologies that the VA and other health care organizations are now implementing to make clinical information more available to patients, and that framing the use of Blue Button separate from those other technologies may be problematic. Further, along with these advances has been discussion about the potential of Blue Button to fuel improvements in health care quality; however, evidence to support these assertions is currently limited.

## Results

### An Evaluation Framework for Blue Button

**Figure 2:**
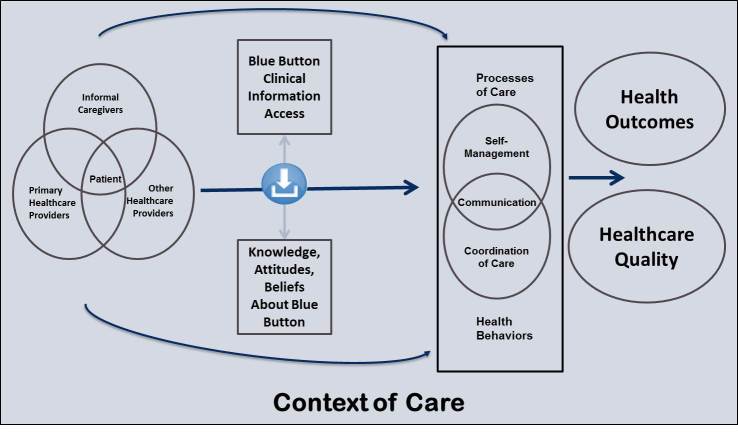
An evaluation framework for Blue Button.

Building on existing models used to inform the evaluation of quality and that emphasize structures, processes, and outcomes at different levels of analysis [40,41], we propose an evaluation framework that examines patient-accessible clinical information technologies (exemplified by Blue Button) not just in terms of a health care organization’s goals, but also focusing on anticipated outcomes for patients and other key stakeholders. Figure 2 presents a framework that depicts how the use of Blue Button can influence processes of care and related behaviors, and ultimately improve outcomes. We describe the framework components below.

### Framework Component 1: Key Stakeholders

A central component of personal health information management is “exchanging” or sharing information in an effort to support health-related tasks, a practice that commonly involves a patient’s informal caregivers (eg, spouses/partners, family members, and others) and their health care providers (eg, primary care doctors, specialists, nurses, and other professionals across health care systems). Previous research has shown that personal health information is often accessed and managed with sharing in mind, and that the exchange of information is performed through multiple means, including paper-based systems and electronic tools [42].

Stakeholders encompass those who use and/or are affected by the clinical information accessed through Blue Button. The proposed framework focuses on patients and three additional stakeholder groups: informal caregivers, health care providers from a patient’s primary health care organization, and other health care providers that a patient may see. As depicted in Figure 2, the patient is at the center of a social system that involves a variety of existing relationships with the other stakeholders who may interact directly with the patient as well as with each other.

### Framework Component 2: Clinical Information Accessible Through Blue Button

Patients can use Blue Button to access different kinds of clinical information either alone or in collaboration with others. They may also share that information with their informal caregivers and health care providers to support various processes of care and associated health behaviors. The knowledge, attitudes, and beliefs that all stakeholders have about Blue Button influence how, why, and if, it is used initially, as well as whether it is adopted and used more routinely.

In the case of the VA, the types of clinical information available through Blue Button were summarized in Table 1. While some evaluation efforts may focus on particular types of information, others may focus on the effects of increased access to clinical information overall.

### Framework Component 3: Blue Button-Sensitive Processes of Care and Associated Health Behaviors

We propose three broad processes of care and associated health behaviors that can be influenced by Blue Button: (1) communication, (2) self-management, and (3) coordination of care. Communication refers to the strategies used to inform and influence individual and community decisions that affect health [43]. Effective communication can increase knowledge and awareness of health issues, shape perceptions, beliefs, and attitudes, reduce barriers, and prompt and sustain behaviors [44]. Clinical information accessed through Blue Button may spur communication, be exchanged through communication, and shape communication in myriad ways. Self-management, in the simplest sense, refers to a patient’s participation in health promotion and/or disease prevention efforts. In many situations, particularly in the context of long-term chronic diseases, the responsibility of managing symptoms, treatment, and other consequences of a condition falls upon the patient, and they must rely on their problem-solving and decision-making skills, their ability to find and use resources, and their relationships with others [45]. Access to and use of clinical information through Blue Button may support a variety of self-management tasks, from monitoring one’s vital signs and related readings to supporting effective management of medications. Finally, coordination of care refers to the usage of services and synchronization of activities among multiple participants in order to facilitate care delivery. Coordination may (or may not) occur among multiple stakeholders in the health care experience, including patients, health care providers, informal caregivers, and others [46]. Ultimately, coordination of care hinges on the effective sharing of clinical information across settings (eg, clinic to clinic, home to clinic) and stakeholders. Returning again to Figure 2, the two overarching arrows convey that stakeholders are confronted with these processes of care and associated health behaviors irrespective of Blue Button; access to clinical information through Blue Button has important potential to influence stakeholder engagement in those processes and behaviors.

### Framework Component 4: Health Care Quality and Health Outcomes

The processes of care described above can influence both health care quality and health outcomes. The Institute of Medicine previously identified six aims for health care improvement, which have since been framed as domains of quality in patient care [5]. They include safety, effectiveness, patient-centeredness, timeliness, efficiency, and equity. Health outcomes pertain to the condition of a patient following some intervention or process, including their degree of wellness and any corresponding needs for care, treatment, or support.

### Patient and Health Services Constructs

The authors of this paper engaged in a structured exercise to identify a thorough set of patient and health services constructs, based on the types of information available through VA Blue Button, which could be used in evaluation efforts. A spreadsheet was distributed that included a list of all VA Blue Button information types (spreadsheet rows) as well as the stakeholder groups of patients, health care providers, and informal caregivers (spreadsheet columns) along with these instructions: “Think of ways that patients, health care providers, and informal caregivers could use the following types of information available through VA Blue Button and in so doing, also reflect on the information required to understand the potential impact of each use.” Each author documented their ideas in their own copy of the spreadsheet, all of which were then collected, reviewed, and deduplicated. The resulting constructs are listed in Table 2 along with a mapping to associated stakeholders. By design, the constructs in the list are untied from the processes of care, associated health behaviors, and outcomes represented in Figure 2, thus providing a high degree of flexibility. Researchers and evaluators can select constructs from this list and combine them in various ways to address the focus of a particular evaluation effort (eg, self-management, communication, coordination of care). In addition, evaluations of Blue Button must use carefully selected measures appropriate for the construct(s) under investigation. Existing measures may be identified in the published literature or novel measures could be designed and validated for a given evaluation.

### Framework Component 5: Context of Care

By context of care, we mean the environment or setting in which patients seek and receive health care services [47]. To the extent that access to and sharing of clinical information through Blue Button is intertwined and contemporaneous with other patient behaviors (eg, use of other PHR features or other information management strategies), other health care services (eg, treatment changes based on clinical care), and other aspects of complexity (eg, socioeconomic issues, personal life changes), teasing out the independent impact of Blue Button will require large studies of considerable power with careful assessment of covariates. Returning to the VA experience, in contrast to other types of organizational delivery models such as fee for service, the VA is structured as a capitated system enabling investments and strategies, which focus on improving the long-term health of patients. The VA also has specialized care systems to meet the needs of Veterans living with prevalent and costly conditions like polytrauma and spinal cord injury. Across these models and systems, the VA also invests in a variety of patient-facing technologies (eg, the My Health***e***Vet PHR portal, mobile applications, telehealth, and kiosks) to facilitate care delivery and address varying Veteran preferences for accessing and receiving services. All of these contextual variables have implications for the ways that Blue Button may be accessed and used. Similarly, the eight million Veterans enrolled for VA services tend to be more complex to manage compared with the general population. Veterans often have less education and lower annual income [48,49], and many have multiple chronic health conditions, a situation that is associated with higher mortality [50]. A substantial number of veterans also use multiple health care systems [51]; and because the VA is a national system, the geographic dispersion of Veterans can create challenges related to access and coordination [52]. Finally, many veterans are faced with unique health care needs that are associated with their military experiences [53-55]. Understanding how such patient population characteristics shape adoption and use of Blue Button is critical.

**Table 2:** Constructs relevant to processes of care and outcomes.

Constructs	Stakeholder		
	Patient	Informal caregiver	Health care provider
Adverse drug interactions	X		X
Allergic events	X		X
Appointment attendance	X		X
Appropriateness of prescriptions	X		X
Caregiver burden		X	
Caregiver capacity to support patient	X	X	
Cholesterol management	X		X
Cross-system information sharing			X
Cross-system medication reconciliation			X
Duplicate services	X		X
Duration/frequency of appointments	X		X
Extent of physical activity	X		
Glucose management	X		X
Medical record accuracy	X		X
Medical record comprehensiveness	X		X
Nutrition management	X		X
Patient activation	X		
Patient attrition	X		X
Patient–caregiver collaboration	X	X	
Patient health perceptions	X		X
Patient–provider communication	X		X
Patient self-monitoring	X		
Patient self-understanding	X		
Preventative self-care practices	X		
Provider time management			X
Provider workload management			X
Quality of care plans	X	X	X
Satisfaction with health care system	X	X	X
Satisfaction with provider–patient interaction	X		X
Service usage (emergency, telephone, urgent care)	X		X
Shared decision-making	X		X
Shared goal setting	X		X
Timeliness of medication refills	X		X
Weight management	X		X

## Discussion

### Principal Findings

Enhancing patient access to clinical information represents a paradigm shift for health care; yet, despite the potential implications of this transformation, there has been little discussion regarding how to systematically evaluate these changes. At present, only isolated reports suggest that patient portals can enhance patients’ access to information, and, in so doing, extend their ability to communicate with providers, support their self-management efforts, and improve coordination of services [16,56-58]. We have provided a framework for the evaluation of patient-accessible clinical information through technology based on VA’s experiences implementing Blue Button. We conclude with a set of seven recommendations relevant across health care organizations and related to future policies and technologies exemplified by Blue Button.

### Policy and Technology Recommendations

####  Blue Button is Best Framed as Part of an Ensemble of Evolving Patient-Facing Technologies

Although seemingly novel at present, Blue Button fits within an expanding array of patient-facing technologies that are now being implemented across health care organizations. Beyond those that are already available, the years ahead will see the proliferation of other patient portal features, mobile applications, and other technologies; all designed to support access to, sharing, and management of clinical information. It is unlikely that patients would choose to use Blue Button to the exclusion of other available technologies; on the contrary, it is more likely that they would use Blue Button in concert with them. In the case of VA, for example, one can easily envision how increased access to clinical information through Blue Button could spur increases in the number of messages exchanged between patients and health care providers using the secure messaging feature of the My Health***e***Vet PHR portal. In this way, use of one technology enhances or “begets” use of another. From this perspective, it may not only be counterproductive to try to untangle and separate use of Blue Button from other technologies, it may also be misleading. As described elsewhere [56], adopting a more complementary vision that situates Blue Button within the milieu of other technologies may more accurately reflect the experiences of patients.

#### Raising Awareness and Educating Stakeholders About Blue Button is a Necessary First Step

In order to rigorously assess the influence of Blue Button on processes of care and outcomes, health care organizations must establish a critical mass of stakeholders who use it. As described earlier, analyzing the independent impact of Blue Button will likely require large, well-powered studies. For this reason, steps must be taken to ensure that patients, their informal caregivers, and their health care providers are using Blue Button to its fullest. We suggest an early investment in research to identify best practices for raising awareness about Blue Button, educating stakeholders about its potential to improve aspects of care, and determining effective strategies for promoting its adoption and sustained use. Later research could then address how best to expand reach of Blue Button to other segments of a patient population, including those with limited Internet access and/or computer skills.

#### Health Care Organizations Must Invest in Data Resources to Support Evaluations of Blue Button

If rigorous evaluations are to be conducted, health care organizations must gather data on use of Blue Button and make those data available for evaluation purposes. Although revisions have recently been made, the policies and terms of use for VA’s My Health***e***Vet PHR portal, for example, historically made data about VA Blue Button use unavailable for research purposes. To fully leverage the data resources that a health care organization has in efforts to understand the effects of Blue Button, appropriate access to individual-level, linkable data is necessary. This includes data regarding when patients have used Blue Button and the types of clinical information accessed, as well as data documenting activities that they have performed using other technologies.

#### Initial Blue Button Evaluations Should Focus on Processes of Care and Associated Health Behaviors

Although the tendency is to evaluate the impact of a novel intervention on outcomes, focusing initial evaluations of Blue Button on processes of care will further our understanding of the role of context and other intervening factors, and reduce the likelihood of producing inaccurate or misleading findings. With this foundation, evaluations can move further along the causal pathway toward outcomes of interest. To that end, we suggest that early evaluations focus on two areas: (1) changes in efficiencies of care, and (2) patient–provider communication during in-person, “brick-and-mortar” clinic visits. For example, reductions in duplicate testing represent a firm example of potential increased efficiencies gained through Blue Button use. Similarly, patients who share clinical information accessed through Blue Button with their health care providers may experience improvements in the accuracy and meaningfulness of their communication.

#### Evaluations of Blue Button Must Account for Unintended Consequences

Also important to acknowledge is that implementation of innovative tools like Blue Button can have unintended consequences. One can speculate, for example, how the interpretation of a prescribed medication list accessed through Blue Button could be difficult for patients and health care providers who do not have access to the various clinical notes that contextualize the medications within the patient trajectory. There may be information missing from a Blue Button report due to the information or timeframe selected by the patient, or because of technical constraints. Exchanging clinical information accessed through Blue Button could potentially result in longer visits as patients present their information and expect health care providers to review it. Moreover, some patients may perceive that they have less privacy and control in light of the ready information access and sharing that Blue Button facilitates. Early evaluations can shed light on such potential unintended consequences and suggest ways to address them through system redesign efforts or targeted interventions

#### Evaluations of Blue Button Must Account for the Complex and Collaborative Nature of Managing Personal Health Information

Implementation of Blue Button represents an early step by health care organizations to support patient access to and exchange of clinical information. A growing body of evidence indicates that the management of personal health information is, in many cases, a collaborative process that involves not only patients, but a variety of other stakeholders [59-61]. Similarly, the ability to move clinical information across organizational boundaries is likely to become an even more pressing need as the complexity of the US health care system increases and consumers seek services across fragmented settings. For these reasons, finding ways to promote effective access to and exchanging of clinical information will be of tremendous importance in the years ahead. There may be considerable value in viewing future evaluations of Blue Button through the lens of collaborative information management, framing it as a kind of social system intervention.

#### Subsequent Research Should Examine Ways to Support Stakeholder Use of Blue Button

As different stakeholders use Blue Button, it is likely that other changes in experience and practice will transpire. Evaluating this cascade of change will be critical. As noted earlier, some patients may find clinical information confusing, and realizing the positive benefits of Blue Button may require additional supportive technologies, translating clinical text into patient terms, providing links to tailored patient education information, and supporting shared decision-making based on the clinical information provided. Thus, in addition to the evaluations we suggest here, considerable basic health informatics research is needed.

### Conclusions

We are just now realizing what was articulated in the medical literature nearly four decades ago [23], “Give the Patient His Medical Record.” Since the release of the pivotal “Crossing the Quality Chasm” report [5], policymakers, clinical administrators, and other stakeholders have envisioned how care could be improved along multiple indicators. The current emphasis on patient engagement coupled with the increase in consumer use of technology [62-64] provides the essential ingredients for ready access to clinical information to support personal health information management and by extension, processes of care and associated health behaviors. However, the evidence base to support Blue Button and related technologies is not yet established. The framework for Blue Button evaluation presented in this paper represents the VA’s early steps along a trajectory of research in this area and will serve as a roadmap to inform the VA’s subsequent evaluation efforts related to this new and important technology.

## Abbreviations

EHR: electronic health records
EKG: electrocardiogram
PDF: portable document format
PHR: personal health records
VA: United States Department of Veterans Affairs
XML: extensible markup language
